# Ruminal metagenomic analyses of goat data reveals potential functional microbiota by supplementation with essential oil-cobalt complexes

**DOI:** 10.1186/s12866-019-1400-3

**Published:** 2019-02-04

**Authors:** Zhaomin Lei, Ke Zhang, Chao Li, Ting Jiao, Jianping Wu, Yubing Wei, Kechuan Tian, Chong Li, Defu Tang, Delmer I. Davis, David P. Casper, Hui Jiang, Xiaolong Wang, Jianfu Wang

**Affiliations:** 1 College of Animal Science and Technology, Gansu Agriculture University, Lanzhou, 730070 China; 2 College of Animal Science and Technology, Northwest A&F University, Yangling, 712100 China; 3 College of Pratacultural Science, Gansu Agriculture University, Lanzhou, 730070 China; 4 Gansu Academy of Agriculture Sciences, Lanzhou, 730070 China; 5 The Animal Husbandry and Veterinary Station of Ganzhou County, Zhangye, 734000 China; 6 Institute of Animal Science, Xinjiang Academy of Animal Science, Urumqi, 830011 China; 7 Ralco Nutrition Inc., Marshall, MN 56258 USA; 8 Furst-McNess Company, Freeport, IL 61032 USA

**Keywords:** Essential oils, Rumen metagenome, Ruminal fermentation, Ammonia emissions, Cobalt

## Abstract

**Background:**

Essential Oils (EO) are complex mixtures of plant secondary metabolites that have been proposed as promising feed additives for mitigating methane and ammonia emissions. We have previously demonstrated that Essential Oil-Cobalt (EOC) supplementation resulted in increased average daily gain and improved phenotypes (cashmere fiber traits, carcass weight, and meat quality) when cashmere goats received supplementation at approximately 2 mg/kg of body weight. However, the ruminal microbiological effects of EO remain poorly understood with regard to the extent to which ruminal populations can adapt to EO presence as feed ingredients. The effects of varying levels of EO require additional study.

**Results:**

In this study, we conducted metagenomic analyses using ruminal fluid samples from three groups (addition of 0, 52, and 91 mg) to evaluate the influence of dietary EOC supplementation on goat rumen bacterial community dynamics. EOC addition resulted in changes of ruminal fermentation types and the EOC dose strongly impacted the stability of ruminal microbiota. The *Bacteroides* sp. and *Succinivibrio* sp. type bacterial community was positively associated with improved volatile fatty acid production when the diet was supplemented with EOC.

**Conclusions:**

A clear pattern was found that reflected rapid fermentative improvement in the rumen, subsequent to butyrate metabolism and EOC based feed additives may affect rumen microbes to further improve feed conversion. This observation indicates that EOC can be safely used to enhance animal productivity and to reduce ammonia and waste gas emissions, thus positively impacting the environment.

**Electronic supplementary material:**

The online version of this article (10.1186/s12866-019-1400-3) contains supplementary material, which is available to authorized users.

## Background

Microbial communities of ruminants are vital for ruminants to access low-quality plant feed, while still producing high-quality protein [1]. The host and its microbiota are two major components that have evolved over millions of years, while ensuring both increased health and opportunities for mutual survival. Although the microbiome community in the rumen is largely stable throughout the entire life of the animal, microbiota diversity and host physiology are largely influenced by the diet (e.g. feed types and composition) [2, 3]. Previous studies have indicated that dietary supplementation of probiotics and prebiotics achieved a positive balance in the gastrointestinal (GI) microbiota of cattle [4].

Essential oils (EO) are volatile aromatic compounds that are produced by plants as complex mixtures of secondary metabolites. EOs contain numerous different chemical substances (20–60 components in each EO) such as alcohols, aldehydes, hydrocarbons, ketones, esters, and ethers [5]. EOs do not exceed a molecular weight of 300, and thus can be physically distinguished from other plant components or membranous tissue [6]. EOs are a “green” choice in nutrition, medicine, and agriculture due to their antibacterial, antiviral, antifungal, anti-nematode, insecticidal, and antioxidant properties. Consequently, anti-fungal and antioxidant activities maintain feed freshness in support of an acceptable level of feed intake. The antimicrobial activity contributes to promoting healthy microbiota population in the lower gut, which directly contributes to gut barrier integrity, reduces inflammation, and improves competitive exclusion of pathogenic bacteria, which leads to a stronger immune system. Recently, several studies have evaluated EOs as natural feed additives to ruminant nutrition and exploited their potential to improve rumen fermentation efficiency [6]. These studies demonstrated the extensive effects of EOs on the EO-type dependence of rumen bacterial communities, particularly of the major families of Prevotellaceae, Lachnospiraceae, and Ruminococcaceae. This result can help to understand the effects of these EOs on rumen digestion and fermentation. However, this study does not describe the changes on ruminal microorganisms and the expression of metabolic pathways and related genes in detail. In addition, the roles of different microbial species in the rumen and their interactions with different doses of EO in vivo remain unknown.

Cobalt (Co) is an activator of various enzymes in the body and is mainly involved in the synthesis of Vitamin B_12_ as Cobalt is a co-factor of Vitamin B_12_. A small amount of cobalt is able to enhance the reproductive capacity of ruminants, while a lack of cobalt often leads to pernicious anemia, dysplasia, low birth rate, reduced milk, weakly born young, and a low rate of weaning survival in livestock [7, 8].

Diets enriched with Oregano Essential Oil (OEO) have a strong anti-oxidant effect that delays lipid peroxidation in the meat during cold storage and cryopreservation. In addition, cryopreservation of meat extends its shelf life [9]. The authors recently reported that dietary supplementation with Essential Oils-Cobalt (EOC) significantly promoted higher average daily gain, while significantly improving fiber quality, carcass weight, and meat quality of goats [9]. We further determined differential expressed genes in skin and liver samples via RNA-seq and found that dietary supplementation of EOC stimulated physiological changes in the animal’s immune system at both physiological and cellular levels [9]. However, whether dietary supplementation of EOC affects the balance of rumen microbiota still remains unknown. Here, a metagenomic analysis was conducted using ruminal fluid samples with the aim to evaluate the influence of dietary EOC supplementation on the bacterial community dynamics of the goat ruminal metabolism. The obtained results will inform the usage of EOC as a feed additive for animal welfare, performance, and economic benefits.

## Results

### Microbial metabolites and morphology in the goat rumen

**Fig. 1:**
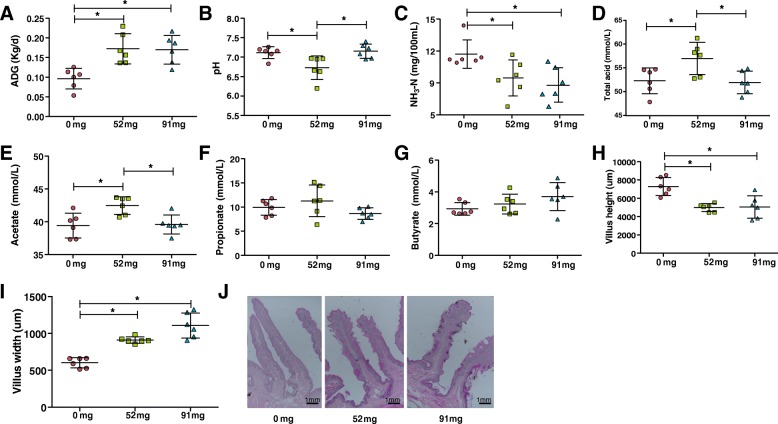
Effects of EOC addition on growth performance and rumen fermentation parameters of goats. Effect of EOC addition on average daily weight (ADG) (kg/d) (**a**), pH in rumen fluid (**b**), ammonia (**c**), total VFA (**d**), acetate (**e**), propionate (**f**), and butyrate (**g**) content of rumen microbial fermentation. Effect of all three treatments on rumen villus height (**h**) and villus width (**i**) (um). **j** H&E staining of rumen villus structure of different groups. * *P* < 0.05

In the present study, slaughter components such as meat quality, carcass weight, dressing percentage, net meat percentage, meat-bone ratio, and perineal fat were found to be significantly different (*P* < 0.05) when comparing the 91 mg group mean value with the control group. The cooked meat percentage was significantly (*P* < 0.05) affected by EOC treatment (Lei et al. 2018). In the present study, all goats had a similar dry matter intake (DMI; see Table 1); however, the average daily gain (ADG) was affected by the feed additive levels. The ADG were significant increased with diet addition EOC (*P* < 0.05; Fig. 1a). In addition, the 52 mg group showed a significant lower pH than other groups (*P* < 0.05; Fig. 1b), suggesting that feed additive levels significantly affect the ruminal pH. It is worth mentioning that the addition of EOC can significantly reduce the production of ammonia (NH_3_-N) in the rumen (*P* < 0.05; Fig. 1c). Ruminal proportions of total acid and acetate were markedly elevated when goats consumed 52 mg EOC (*P* < 0.05; Fig. 1d & e). Acetate is mostly absorbed through the ruminal wall, transported unmodified into the blood, then transported to the liver, and moved via the portal blood to peripheral tissues for oxidation via tricarboxylic acid to gain energy or for fatty acid synthesis. Propionate and butyrate also showed a similar increasing trend; however, this difference was not significant (*P* > 0.05; Fig. 1f & g). In summary, the volatile fatty acid (VFA) content is one of the main indicators for ruminal fermentation, while the main impact of fundamental factors is the structure of the diet. These results indicate that adding 52 mg EOC changed in ruminal fermentation types.

**Table 1:** Ingredients and nutrients of the experimental diet %

Ingredients	Proportion %
Corn cob	12.00
Alfalfa	5.00
Rape straw	10.00
Wheat straw	10.00
Corn	48.60
Soybean meal	2.00
Rape seed meal	3.00
Cotton seed meal	6.00
Premix	1.00
Stone powder	0.90
NaCl	0.50
Baking soda	1.00
Total	100.00
Nutrient level	
Digestible energy (MJ/kg)	11.20
Crude protein (%) of DM	10.50
NDF (%)	38.40
Starch (%)	29.50
Ca (%)	0.58
P (%)	0.29

Note: The first stage consisted of dry matter intake of 800 g; the second stage consisted of dry matter intake 900 g; the third stage consisted of dry matter intake of 1000 g. Formulated to provide (per kg of dry matter): S, 200 mg; Fe, 25 mg; Zn, 45 mg; Cu, 8 mg; Mn, 40 mg; I, 0.3 mg; Se, 0.2 mg; Co,0.1 mg; VA, 980 IU; VD, 120 IU; VE, 25 IU

The villus height and width parameters were used to determine the effect of EOC supplementation on ruminal morphology. The villus height in the 0 mg group significantly enhanced its growth in the dorsal rumen (*P* < 0.05; Fig. 1h). With regard to the villus width, 91 mg EOC diet significantly enhanced its value in the dorsal ruminal sac (*P* < 0.01; Fig. 1i), suggesting that an EOC enriched diet tended to stimulate ruminal villus development. Moreover, H&E staining was used to examine the ruminal morphology and the results showed that an EOC enriched diet significantly improved the function of ruminal nutrient digestion and subsequent absorption (Fig. 1j).

### Ruminal microbial community composition in goats

**Fig. 2:**
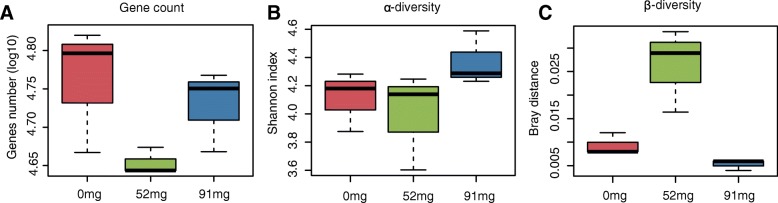
Comparison between shotgun sequencing data of rumen content from the three EOC treatments groups. **a** Box plot of the gene count in three EOC treatments groups. **b** α-diversity Shannon index (**c**) and β-diversity (Bray-Curtis similarity index)

To illustrate whether the VFA and NH_3_-N values were associated with the variation of rumen microbiota that may result from the addition of EOC, we conducted metagenomic analyses using rumen fluid samples from different EOC treatment groups. Deep sequencing of rumen fluid DNA samples generated 107 Gb of high-quality data with an average of 11.84 Gb per sample (Additional file 1: Table S1), enabling identification of 699,497 non-redundant (NR) genes with an average N50 contig length of 2.83 Kb. The EOC enriched diet group had lower gene counts than controls (Fig. 2a) and the 52 mg EOC group had lower bacterial diversity than controls (Fig. 2b), indicating that gene richness was lower in the EOC diet group. We observed higher β diversity in the 52 mg EOC diet group, when the supplementary of EOC reached 91 mg, we observed lower β diversity than controls, indicating the dose of EOC significantly influences the community structure in rumen (Fig. 2c).

**Fig. 3:**
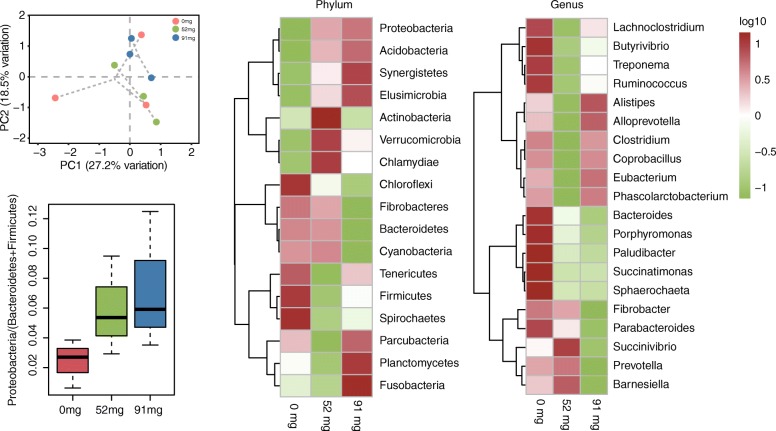
Effects of three EOC treatments on the composition of goat rumen microbiota. **a** PCA analysis of the UniFrac distance in the rumen content of three EOC treatment groups. **b** Heatmaps representing microbiotas in the phylum level from the three EOC groups. **c** Heatmaps represent top 20 microbiotas in the genus level from the three EOC groups. **d** Box plot of the Proteobacteria / (Bacteroides + Firmicutes) to evaluate whether the GI microbiology community was imbalanced in three EOC treatments

Of the 17 identified phyla, *Bacteroidetes* and *Firmicutes* were predominant in goat rumen samples (Additional file 1: Table S2). However, with increased EOC dose, an increase in the number of *Proteobacteria* was found, while an increase in the number of *Acidobacteria*, *Synergistetes*, and *Elusimicrobia* was also found. Compared to the 0 mg group and 52 mg group, decreases in *Firmicute*s were observed in the rumen samples. Compared to the 52 mg group and 91 mg group, significant decreases in *Tenericutes* were observed in the rumen samples (*P* = 0.001; Fig. 3b). At the genus level, most of the annotated genes belonged to *Prevotella*, followed by *Bacteroides*, *Alistipes*, *Treponema*, *Clostridium*, and *Ruminococcus* in the 0 mg supplement group (Additional file 1: Table S3). Compared to the 0 mg group and 52 mg group, significant changes in *Succinimonas*, *Brachyspira*, *Burkholderia*, and *Staphylococcus* were found (*P* < 0.05). Compared to the 52 mg group and 91 mg group, *Prevotella* significantly decreased in the ruminal samples (*P* = 0.009) (Fig. 3c). The microbiome abundance of metagenomics in the genus obtained via principal component analysis (PCA) of each group using the Bray–Curtis similarity metric showed that the bacterial populations in each sample were best clustered together according to the differences in EOC content in the diet. These three groups showed distinct separation and the spatial location of 0 mg and 52 mg groups was dissimilar, indicating that the microbiota structure was different in both groups (Fig. 3a). These results suggest that the increase of EOC dosage has a stronger impact on the goat rumen microbiota, especially on the lower abundance of bacteria (Fig. 3c). Our results suggested that the addition of EOC did not significantly affect the abundance of methanogenic bacteria in the rumen (Additional file 1: Table S4).

### Association of rumen microbial with metabolites

**Fig. 4:**
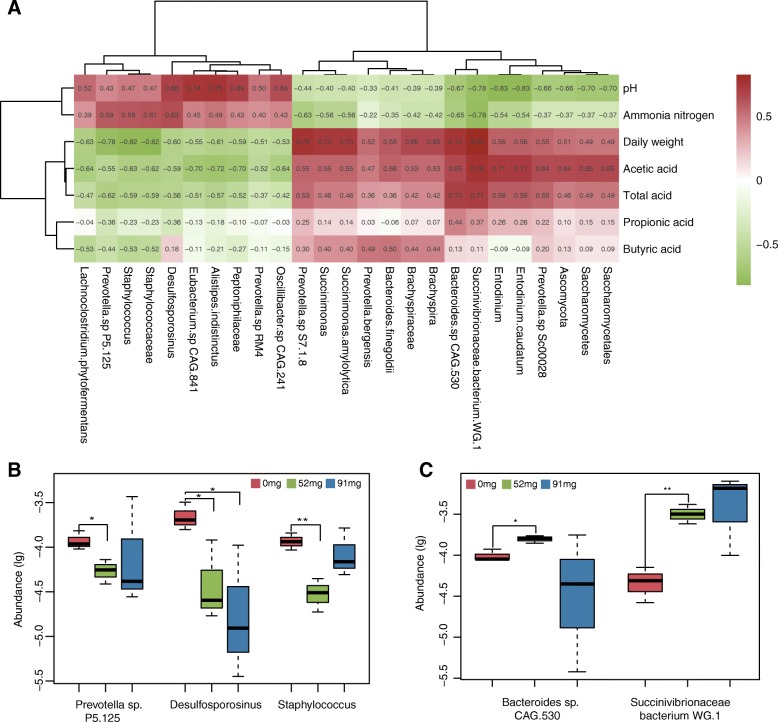
Correlations between production variables and relative taxa abundance. **a** Pearson’s correlation of the dominant bacteria across rumen samples. All presented correlations were statistically significant (*P* < 0.05), with strong correlations and weaker correlations indicated via shade. The scale colors denote whether the correlation was positive (closer to 1, green) or negative (closer to − 1, red) between the taxa and production variables. **b** & **c** Bacterial distribution in 0 mg, 52 mg and 91 mg EOC groups

To determine the correlation between metabolites and changes of rumen microbiome in different groups, we conducted association analyses (Pearson’s rank correlation coefficients) of the phenotypic module to detect significantly different microbiome. Furthermore, pH, NH_3_-N, daily weight, acetic acid, total acid, propionic acid, and butyric acid correlated with alterations in the rumen microbiota. The authors found that *Desulfosporosinus*, *Staphylococcus*, and *Prevotella* sp. were positively correlated with the production of NH_3_-N and were negatively correlated with the production of VFA (Fig. 4a). In addition, we also conducted a difference analysis on the above genus and found a significant difference between the EOC diet group and the control (Fig. 4b). We furthermore found that *Bacteroides* sp. and *Succinivibrio* sp. were positively correlated with VFA production and negatively correlated with NH_3_-N production (Fig. 4a). The abundance of *Bacteroides* sp. and *Succinivibrio* sp. were significantly lower compared to the 0 mg group (Fig. 4c), indicating that these species may constitute potential linking ruminal microbiota and metabolic status.

### Functional characterization of the EOC diet group microbiome

**Fig. 5:**
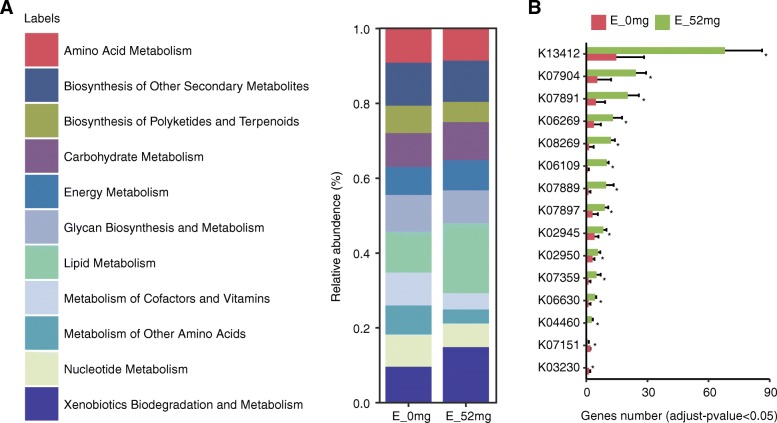
Majority of the gene sequences annotated to KEGG, representing the functional composition of the rumen bacterial microbiota in different EOC treatment groups. **a** Functional enrichment of rumen microbiota in different EOC treatments. **b** Distinct KOs assigned to enzymatic reactions involved in different metabolism with amino acid levels

The gene set enrichment analysis (GSEA) [10] was used to identify differentially abundant Kyoto Encyclopedia of Genes and Genomes (KEGG) pathways in metagenome datasets. We identified 2130 KEGG Orthology (KOs) present in nine samples, of which 246 KOs differed in both the 0 mg group and 52 mg group (FDR < 0.05; Additional file 1: Table S5). We focused on selecting subsets of genes that corresponded to EOC supplementation. Significant gene categories enriched the carbohydrate metabolism, lipid metabolism, and xenobiotics biodegradation (Fig. 5a). For both genes, significant differences were found between the total number of reads mapped to each gene between the 0 mg group and the 52 mg group (*P* ≤ 0.05; Additional file 1: Table S6). The most highly expressed genes in the 52 mg group were associated with calcium-dependent protein kinase (K13412), Ras-related protein Rab-11A (K07904), Ras-related protein Rab-22 (K07891), serine/threonine-protein phosphatase PP1 catalytic subunit (K06269), and serine/threonine-protein kinase ULK/ATG1 (K08269; Fig. 5b). In summary, these results demonstrate that the microbiota of the EOC diet group may have a higher capacity for nitrogen utilization and a higher capacity for xenobiotic biodegradation and metabolism.

## Discussion

The diversity of microbiome in the rumen plays a central role to ensure the stability of the rumen ecosystem, while improving its ability to adapt to a wide range of dietary management strategies. EO contains many different chemical substances including alcohols, aldehydes, hydrocarbons, ketones, esters, and ethers [11]. Adding EO for in vitro rumen microbial fermentation, led to phenolic compounds exhibiting antimicrobial activities mainly by reducing the diet ferment ability and by shifting the VFA pattern towards less propionate and more butyrate [11]. In addition, OEO mainly consists of phenolic resin monoterpenoids known as thymol and carvacrol, achieving strong and broad spectrum antibacterial activity due to the presence of hydroxyl groups [12].

The obtained results suggest that the EOC dose strongly impacts the stability of rumen microbiota and that the dose of the additive can cause rapid changes *of Proteobacteria* in the rumen. Members of *Prevotella*, a Gram-negative genus, increased their population in response to EOC addition; however, increasing the added dose of EOC decreased their population. On the other hand, members of the *Firmicutes*, a largely Gram-positive phylum, were decreased in both EOC diet groups, and bacterial groups in the genus *Clostridia*, which contains most of the Gram-positive rumen bacteria, were decreased in response to EOC addition. However, addition of other oils (i.e., peppermint oil) did not cause these changes of bacteria [13]. Evidently, effects of EOC on rumen bacteria are both species and EO-type dependent.

In addition, members of the *Acidobacteria*, an acidophilic bacterium, increased their population in response to EOC addition; at present, there is no clear conclusion about the ecological function of *Acidobacteria*. Only few strains can be cultivated independently; therefore, we can only summarize and speculate based on the existing research. Environmental factors significantly affected the abundance of *Acidobacteria*, the most influential of which is the pH [14]. Previous isolation and culture studies showed that the *Acidobacteria* strains KBS83 and KBS96 have cellulolytic ability [15, 16]. Studies have also shown that *Acidobacteria* accounts for more than 50% of the soil microbial community. For example, the microbial community of soil samples around chestnut tree roots consisted of as much as 65% *Acidobacteria* [17]. *Acidobacteria* are widely distributed in the natural environment and even in extreme environments [18], such as polluted environments [19] and waste-water environments [20]; therefore, it has been speculated that *Acidobacteria* exerts a driver role in different ecosystem.

Hyper-Ammonia-Producing (HAP) bacteria are the most sensitive rumen bacteria in pure culture and can produce NH_3_ from amino acids. Several steps have been reported to be involved in the catabolism of all proteins involved in *Prevotella* sp. [21]. Previous studies have shown that EO inhibits the growth of certain HAP bacteria (e.g. *Clostridium sticklandii* and *Peptostreptococcus anaerobius*), while other HAP bacteria (e.g. *Clostridium aminophilum*) are not sensitive. Low numbers of HAP bacteria have been reported for the rumen (1% rumen bacterial population); however, they have high deamination [22, 23]. Our study showed that addition of EO largely affected the proportion of low abundance bacteria in the rumen. The bacteria associated with NH_3_-N production were significantly different between the 52 mg and control groups. Moreover, the phenotype data suggested that the addition of EO increases the production of acetate and butyrate. This contributed to the increase of the total volatile acid production. Another study of in vitro ruminal microbial fermentation suggested that EO exhibited antimicrobial activities mainly by reducing fermentation and by shifting the VFA pattern towards less propionate and more butyrate [11]. In general, the most fundamental role of VFA in ruminants is to provide energy and the main role of ruminal microorganisms is to decompose nutrients into VFA and ammonia, and then use this for re-biosynthesis or for the energy metabolism. Acetate is absorbed by the ruminal wall, and most of the unmodified acetate enters the portal blood, is transported to the liver, and subsequently to peripheral tissues, where it is used for oxidation by tricarboxylic acid for cellular energy or fatty acid synthesis. Three-carbon propionate is the main precursor of gluconeogenesis and as a 3-carbon compound, it is considered a glucose former (six carbons). Butyrate (most of the conversion to β-hydroxyl butyrate) is exposed to several types of body tissues in the rumen, such as the reticulum wall that support the absorption process and is an especially important muscle energy source. In addition, previous studies reported that addition of EO reduced the methane production [5]. EO can either directly inhibit methanogenic archaea or indirectly decrease methane production via direct depression [24]. EOs may cause changes in the archaeal community structure or in the activity of the methanogenesis pathway, thus subsequently decreasing methanogen abundance and methane production [25]. Our study found that EOC did not significantly affect the abundance of methanogenic bacteria.

The proteobacterial load has been suggested as a potential diagnostic criterion for dysbiosis and disease, and its ratio belongs to the Proteobacteria / (Bacteroides + Firmicutes) ≥ 0.19 and has been used as a marker of whether the GI microbiology community is imbalanced [26, 27]. Here, the metagenomic sequencing results is summarized and the ratio of Proteobacteria / (Bacteroides + Firmicutes) was below 0.19 in the EOC diet groups (Fig. 3d); therefore, the addition of EOC did not cause dysbiosis of the goat ruminal microbiology. Via correlation analysis, we found that *Bacteroides* spp. and *Succinivibrio* sp. follow a positive correlation with the production of VFA. According to previous studies, we found that an environment favoring large numbers of *Succinivibrio* sp. would not only contribute to substrate oxidations and reductions, but closely coupled to this, with little methane being formed, it would also ensure that more digestible energy is available for the host animal [28]. Addition of EOC significantly increased the abundance of *Succinivibrio* sp. The increased abundance of *Bacteroides* spp. and *Succinivibrio* sp. encourages more carbohydrates to be broken down into VFAs for the body’s energy and for improved feed utilization.

## Conclusions

In summary, the metagenome data that was analyzed in this study provides strong evidence that a *Bacteroides* spp. and *Succinivibrio* sp. type bacterial community was associated with the production of VFA as a result of EOC supplementation to the feed diet. A clear pattern was found, reflecting rapid fermentative growth in the rumen subsequent metabolism to butyrate. EOs may act as promising natural substances to mitigate ruminal methane and ammonia production, improve rumen fermentations, and thus, reduce the environmental impact of ruminant production.

## Methods

### Animals and sampling

All animals used in this study have been described previously [29]. Briefly, a total of 45 castrated male cashmere goats from the experimental station of Gansu Agriculture University (Pingshanhu, Zhangye, Gansu, China) were randomly divided into intact designations of Rum-A-Fresh™ (RAF; three groups). These animals were raised at the university farm. EO and Co were added as Rum-A-Fresh™ with an organic cobalt level of 0.75% in the RAF product. The amount of EO is 1.3% of the RAF product. For each group, 0, 52, and 91 mg per head/day of EOC were added to the daily diet in the form of an EO, lactic acid, and cobalt carbonate with clinoptilolite used as an ingredient dispersant. Three goats per group were randomly selected and were slaughtered at day 90 in an accredited abattoir, using carbon dioxide to stun the animal. The rest of animals in each group were continually raised for other experimental purposes. Ruminal fluid samples were collected and then separated into solid and liquid fractions via filtering through a muslin cloth. Each sample was immediately frozen in solid carbon dioxide and then transported to the laboratory where samples were stored at − 80 °C prior to analyses. Parts of dorsal rumen were used for hematoxylin-eosin (H&E) staining, following the H&E staining procedures according to the method described by Carter et al. [30]. Parts of the dorsal rumen were extracted from different goats and placed in tubes containing 4% paraformaldehyde solution (made with 0.1 M sodium phosphate buffer, pH = 7.4). The following steps were embedding, cutting into slices, baking slides, H&E staining, and mounting [31]. The pH of the ruminal fluid was measured immediately after collection by a mobile pH meter. Volatile fatty acid (VFA) concentrations were determined using a gas chromatograph (Agilent 7890A, China) according to the method of Hoskin et al. [32]. NH_3_-N levels were analyzed with a colorimetric technique using a spectrophotometer (721–100, China) following the method of Chaney [33].

### DNA extraction, PCR amplification, and metagenomic sequencing

Microbial DNA was extracted from rumen fluid samples using the TIANGEN kit (TIANGEN Biotech Co., Ltd., Beijing, China) according to the manufacturer’s instructions. All samples were sequenced on the Illumina HiSeq 4000 platform (with an insert size of 300 bp and a read length of 125 bp) at the Bioinformatics GmbH (Novogene), after quality control and read-alignment to the goat genome. The remaining high quality reads were used for further analysis [34].

The assembly of reads was conducted using Short Oligonucleotide Analysis Package (SOAP) denovo [35]. For each sample, a series of k-mer = 39 (parameters: -d 1, −R, −u) was used [36, 37]. The assembled scaffolds are disrupted from the N junction, resulting in an N-free sequence fragment. Clean data of each quality control was compared with Soap Aligner software to assembled Scaftigs of each sample to obtain unused PE reads (parameters: -u, − 2, −m 200) [36]. The best samples with the longest N50 of the rest of the stent were selected. Unused readings for each sample were assembled using the same parameters. The MetaGeneMark predictor gene was used for shafting of more than 500 bp (prokaryotic GeneMark.hmm version 2.10). Then, a non-redundant gene catalog constructed CD-HIT (parameter: - 0 - 0.9 - 1 - 0 - G D C 0.9) using the 0.9 sequence with the lowest cutoff for the shorter sequence coverage 0.9 cutoff [38].

To determine gene abundance, the gene catalog was read and readjusted with the soap2 usage parameter: M 200 × 600–255. Only the gene ≥2 map was considered present in the sample. Gene abundance was calculated via the number of reads and normalized via gene length [36].

### Taxonomic annotation and abundance profiling

To evaluate the classification task, the genetic permutation combination NR database using diamond was utilized (version 0.8.24.86 with default parameter values, except ≤1e-5) [39]. As previously described [40], for each gene, a major reserved game (which is defined by the 10 × e value of the energy value ≤ the most popular definition) was used to distinguish between taxa. The level of classification of each gene was determined in Megan and implemented with the lowest common ancestral algorithm [41]. The abundance of a taxonomic group was calculated via the sum of genes of a feature of the annotation.

### Species and functional annotation

Uni-genes were compared to NCBI’s NR (version: 2016-11-05) database using the DIAMOND software pair (blastp, e-value ≤1e-5) [39]. For the alignment of each sequence, the authors selected the e-value ≤ minimum e-value × 10 for subsequent analysis [40]. After filtering, and since there may be multiple alignment results for each sequence, multiple different species classification information was obtained. To ensure biological significance, the LCA algorithm (system classification applied to MEGAN software) used a branch before the classification level, as the sequence of species annotation information [42]. All genes available in the gene research catalogue were aligned to the KEGG database (Release 73.1, with animal and plant genes removed). Abundance of the KEGG orthologue/module was calculated by summing the abundance of genes that were annotated to the same feature. Metagenome sequences generated were entered into the National Center for Biotechnology Information (NCBI) under accession numbers SRA: SRP149856.

### Statistical analysis

The Chao index was calculated at the genera level with Vegan R packages in R software (version 3.2.4). PCA was analyzed using the FactoMine R package in R software (version 3.2.4). Differential abundance of genes, phylum, genus, species, and KO modules was tested using the Student’s t-test, and *P* values were corrected for multiple testing using the Benjamin & Hochberg method.

## Additional file

Additional file 1: **Table S1.** Description of the assembly data of the nine samples. **Table S2.** Comparison of the phyla in rumen bacteria of three treatment groups. **Table S3.** Comparison of the genera in rumen bacteria of three treatment groups. **Table S4.** Comparison of the change in rumen methanogens of three treatment groups. **Table S5.** Comparisons of the functions of the rumen bacterial microbiota in the three treatment groups. **Table S6.** EOC-associated KO pathways. (PDF 381 kb)

## Abbreviations

ADG: Average daily gain
Co: Cobalt
DMI: Dry matter intake
EO: Essential Oils
EOC: Essential Oil-Cobalt
GI: Gastrointestinal
HAP: Hyper-Ammonia-Producing
KEGG: Kyoto Encyclopedia of Genes and Genomes
KOs: KEGG Orthology
NCBI: National Center for Biotechnology Information
NH_3_-N: Ammonia
NR: Non-redundant
OEO: Oregano Essential Oil
PCA: Principal component analysis
VFA: Volatile fatty acid
