# Supplementary material for: Ruminal metagenomic analyses of goat data reveals potential functional microbiota by supplementation with essential oil-cobalt complexes
Source: BMC Microbiol. 2019 Feb 4;19:30. doi: 10.1186/s12866-019-1400-3 (PMC6362596; doi:10.1186/s12866-019-1400-3)
Supplement: Supplementary file 1 — Table S1. Description of the assembly data of the nine samples. Table S2. Comparison of the phyla in rumen bacteria of three treatment groups. Table S3. Comparison of the genera in rumen bacteria of three treatment groups. Table S4. Comparison of the change in rumen methanogens of three treatment groups. Table S5. Comparisons of the functions of the rumen bacterial microbiota in the three treatment groups. Table S6. EOC-associated KO pathways. (PDF 381 kb) [file 12866_2019_1400_MOESM1_ESM.pdf]

Supplementary Material：

Ruminal metagenomic analyses of goat data reveals potential functional microbiota by supplementation with essential oil-cobalt complexes

Zhaomin Lei[1], Ke Zhang[2], Chao Li[2], Ting Jiao[3], Jianping Wu[4], Yubing Wei[5], Kechuan Tian[6], Chong Li[1], Defu Tang[1], Delmer I Davis[7], David P Casper[8], Hui Jiang[1], Xiaolong Wang[2], Jianfu Wang[1]*

[1]College of Animal Science and Technology, Gansu Agriculture University, Lanzhou, 730070, China.

[2]College of Animal Science and Technology, Northwest A&F University, Yangling, 712100, China.

[3]College of Pratacultural Science, Gansu Agriculture University, Lanzhou, 730070, China.

[4]Gansu Academy of Agriculture Sciences, Lanzhou, 730070, China.

[5]The Animal Husbandry and Veterinary Station of Ganzhou County, Zhangye, 734000, China.

[6]Institute of Animal Science, Xinjiang Academy of Animal Science, Urumqi, 830011, China.

[7]Ralco Nutrition Inc., Marshall, MN, 56258, USA.

[8]Furst-McNess Company, Freeport, IL 61032 USA.

*Correspondence: wangjf@gsau.edu.cn

Running title: Functional microbiota by supplementation with essential oil-cobalt complexes

E-mails: Zhaomin Lei: leizm@gsau.edu.cn

Ke Zhang: kezhangdk@163.com

Chao Li: lichao_xinong@163.com

Ting Jiao: Jiaoting207@126.com

Jianping Wu: wujp@gsagr.ac.cn

Yubing Wei: Weiyb258@163.com

Kechuan Tian: tiankechuan@163.com

Chong Li: lichong@gsau.edu.cn

Defu Tang: tangdf@gsau.edu.cn

Delmer I Davis: del.davis@ralconutrition.com

David P Casper: david.casper@mcness.com

Hui Jiang: 1353090083@qq.com

Xiaolong Wang: xiaolongwang@nwafu.edu.cn

Jianfu Wang: wangjf@gsau.edu.cn

Supplementary table 1. Description of the assembly data from the 9 samples

	Mean ± SD	Sum
High quality data	11.84 ± 1.92 Gb	107 Gb
Assembled scatigs length	80.57 ± 17.46 Mb	725.13 Mb
N50	2,823 bp	25,409 kb
ORFs number	77,722 ± 14,026	699,497

Supplementary Table S2. Comparison of the phylain rumen bacterial of three treatment group.

	0mg			52mg			91mg			T-test		
	0-1	0-2	0-3	52-1	52-2	52-3	91-1	91-2	91-3	0_52	0_91	52_91
Bacteroidetes	0.4081	0.2178	0.3519	0.2979	0.2508	0.4142	0.2640	0.2253	0.1533	0.9497	0.1611	0.1416
Firmicutes	0.1260	0.1323	0.0363	0.0225	0.0465	0.0630	0.0823	0.0719	0.0385	0.1773	0.3702	0.3166
Proteobacteria	0.0206	0.0095	0.0025	0.0172	0.0282	0.0139	0.0432	0.0105	0.0113	0.2614	0.4184	0.8775
Tenericutes	0.0019	0.0003	0.0001	0.0002	0.0001	0.0001	0.0004	0.0005	0.0005	0.3311	0.6335	0.0018
Spirochaetes	0.0018	0.0347	0.0011	0.0003	0.0015	0.0044	0.0085	0.0017	0.0014	0.4022	0.4865	0.5343
Verrucomicrobia	0.0018	0.0020	0.0004	0.0044	0.0007	0.0018	0.0016	0.0006	0.0033	0.5242	0.6807	0.7678
Actinobacteria_<phylum>	0.0014	0.0011	0.0005	0.0008	0.0006	0.0016	0.0011	0.0010	0.0009	0.9461	0.9949	0.9260
Chlamydiae	0.0008	0.0005	0.0002	0.0002	0.0009	0.0018	0.0014	0.0002	0.0005	0.4359	0.6644	0.6937
Fibrobacteres	0.0007	0.0112	0.0011	0.0050	0.0031	0.0007	0.0003	0.0000	0.0002	0.7163	0.2888	0.0889
Cyanobacteria	0.0007	0.0003	0.0002	0.0001	0.0003	0.0008	0.0003	0.0003	0.0004	0.9922	0.7136	0.7233
Chloroflexi	0.0006	0.0001	0.0004	0.0001	0.0001	0.0003	0.0001	0.0000	0.0002	0.3033	0.1876	0.4428
Planctomycetes	0.0004	0.0005	0.0001	0.0001	0.0001	0.0005	0.0004	0.0001	0.0010	0.5856	0.5514	0.3647
Fusobacteria	0.0004	0.0001	0.0001	0.0000	0.0001	0.0003	0.0005	0.0007	0.0002	0.7041	0.1601	0.0903
Parcubacteria	0.0003	0.0002	0.0001	0.0000	0.0000	0.0002	0.0003	0.0001	0.0003	0.2889	0.6624	0.2013
Acidobacteria	0.0002	0.0000	0.0000	0.0000	0.0000	0.0006	0.0003	0.0002	0.0002	0.5546	0.0475	0.7903
Synergistetes	0.0001	0.0001	0.0001	0.0000	0.0003	0.0007	0.0002	0.0005	0.0021	0.2471	0.2028	0.3561
Elusimicrobia	0.0000	0.0001	0.0000	0.0000	0.0015	0.0001	0.0002	0.0064	0.0001	0.3700	0.3503	0.4720

Supplementary Table S3. Comparison of the genera in rumen bacterial of three treatment group.

	0 mg			52 mg			91 mg			T-test		
taxa	0-1	0-2	0-3	52-1	52-2	52-3	91-1	91-2	91-3	0_52	0_91	52_91
Staphylococcus	0.0001	0.0001	0.0001	0.0000	0.0000	0.0000	0.0002	0.0000	0.0001	0.0066	0.5772	0.1594
Brachyspira	0.0000	0.0000	0.0000	0.0001	0.0001	0.0001	0.0002	0.0003	0.0002	0.0162	0.0017	0.0218
Succinimonas	0.0003	0.0003	0.0000	0.0007	0.0014	0.0009	0.0018	0.0001	0.0003	0.0202	0.3688	0.6734
Desulfosporosinus	0.0002	0.0003	0.0002	0.0000	0.0000	0.0001	0.0001	0.0000	0.0000	0.0421	0.0329	0.7809
Pseudobutyrivibrio	0.0003	0.0003	0.0002	0.0001	0.0001	0.0002	0.0001	0.0002	0.0000	0.0666	0.0644	0.5252
Lachnoclostridium	0.0017	0.0012	0.0010	0.0003	0.0007	0.0010	0.0006	0.0019	0.0005	0.0689	0.5714	0.5380
Anaerotruncus	0.0008	0.0005	0.0002	0.0000	0.0001	0.0001	0.0011	0.0001	0.0002	0.0758	0.9074	0.2770
Acidiphilium	0.0003	0.0006	0.0001	0.0000	0.0000	0.0000	0.0014	0.0001	0.0002	0.0880	0.6422	0.2601
Butyrivibrio	0.0041	0.0030	0.0012	0.0005	0.0014	0.0008	0.0014	0.0020	0.0007	0.0982	0.2027	0.3603
Selenomonas	0.0004	0.0001	0.0002	0.0003	0.0052	0.0073	0.0021	0.0005	0.0006	0.1267	0.1863	0.2135
Megamonas	0.0000	0.0000	0.0000	0.0000	0.0002	0.0003	0.0001	0.0000	0.0000	0.1278	0.5850	0.1460
Megasphaera	0.0000	0.0001	0.0000	0.0000	0.0003	0.0003	0.0001	0.0003	0.0001	0.1292	0.1899	0.7355
Coprococcus	0.0006	0.0002	0.0002	0.0001	0.0001	0.0000	0.0001	0.0000	0.0001	0.1392	0.1538	0.6383
Tolumonas	0.0000	0.0001	0.0000	0.0001	0.0009	0.0004	0.0000	0.0000	0.0000	0.1542	0.5983	0.1324
Clostridium	0.0128	0.0072	0.0026	0.0014	0.0020	0.0036	0.0065	0.0116	0.0032	0.1582	0.9156	0.1324
Mitsuokella	0.0003	0.0000	0.0000	0.0002	0.0006	0.0011	0.0005	0.0000	0.0001	0.1635	0.6731	0.2379
Chryseobacterium	0.0000	0.0001	0.0001	0.0001	0.0002	0.0004	0.0001	0.0003	0.0001	0.2151	0.1365	0.7479
Aeromonas	0.0000	0.0002	0.0000	0.0000	0.0007	0.0005	0.0000	0.0002	0.0000	0.2153	0.8760	0.1903
Pedosphaera	0.0000	0.0002	0.0000	0.0000	0.0000	0.0000	0.0002	0.0001	0.0014	0.2170	0.2726	0.2226
Azospirillum	0.0000	0.0002	0.0001	0.0000	0.0000	0.0000	0.0003	0.0003	0.0006	0.2172	0.0707	0.0239
Ruminobacter	0.0000	0.0002	0.0000	0.0087	0.0015	0.0008	0.0190	0.0010	0.0012	0.2281	0.3044	0.6243
Paenibacillus	0.0011	0.0005	0.0002	0.0001	0.0004	0.0002	0.0005	0.0002	0.0003	0.2524	0.4323	0.2864
Lactobacillus	0.0001	0.0001	0.0002	0.0001	0.0000	0.0001	0.0003	0.0004	0.0001	0.2558	0.2381	0.1177
Peptoclostridium	0.0003	0.0001	0.0001	0.0001	0.0001	0.0001	0.0003	0.0002	0.0003	0.2677	0.2504	0.0057
Streptomyces	0.0000	0.0000	0.0000	0.0001	0.0001	0.0000	0.0002	0.0000	0.0002	0.2740	0.0814	0.2195
Acidaminococcus	0.0001	0.0009	0.0001	0.0001	0.0001	0.0000	0.0001	0.0000	0.0001	0.2762	0.3168	0.5883
Corallococcus	0.0000	0.0003	0.0000	0.0000	0.0000	0.0000	0.0001	0.0000	0.0000	0.2769	0.5045	0.0378
Roseburia	0.0003	0.0007	0.0001	0.0000	0.0001	0.0003	0.0003	0.0002	0.0001	0.2933	0.4556	0.4379
Coprobacillus	0.0049	0.0006	0.0005	0.0005	0.0000	0.0001	0.0013	0.0033	0.0015	0.2941	0.9882	0.0500
Alistipes	0.0111	0.0082	0.0077	0.0013	0.0043	0.0107	0.0192	0.0099	0.0049	0.2962	0.6157	0.3056
Ruminococcus	0.0032	0.0162	0.0009	0.0004	0.0009	0.0018	0.0041	0.0022	0.0012	0.2966	0.4247	0.1971
Ruminiclostridium	0.0015	0.0004	0.0002	0.0001	0.0001	0.0004	0.0004	0.0006	0.0003	0.3042	0.5730	0.1332
Coraliomargarita	0.0001	0.0001	0.0001	0.0014	0.0002	0.0001	0.0002	0.0001	0.0003	0.3052	0.4372	0.3558
Fretibacterium	0.0000	0.0000	0.0000	0.0000	0.0000	0.0002	0.0001	0.0002	0.0010	0.3059	0.2030	0.2700

Acholeplasma	0.0012	0.0001	0.0000	0.0000	0.0000	0.0000	0.0003	0.0003	0.0001	0.3101	0.5929	0.0160
Pedobacter	0.0000	0.0001	0.0000	0.0001	0.0001	0.0003	0.0004	0.0001	0.0003	0.3243	0.1188	0.3263
Sphaerochaeta	0.0003	0.0064	0.0001	0.0000	0.0000	0.0002	0.0001	0.0000	0.0000	0.3494	0.3466	0.8345
Paludibacter	0.0026	0.0014	0.0005	0.0017	0.0001	0.0001	0.0000	0.0015	0.0001	0.3509	0.2974	0.9198
Butyricimonas	0.0008	0.0002	0.0003	0.0000	0.0003	0.0003	0.0004	0.0004	0.0003	0.3566	0.7016	0.2452
Anaerovorax	0.0000	0.0016	0.0000	0.0000	0.0000	0.0000	0.0001	0.0002	0.0000	0.3655	0.4585	0.1853
Proteiniphilum	0.0005	0.0000	0.0001	0.0000	0.0001	0.0001	0.0001	0.0001	0.0001	0.3668	0.5012	0.3993
Oribacterium	0.0003	0.0013	0.0013	0.0006	0.0009	0.0004	0.0005	0.0012	0.0007	0.3693	0.7014	0.4653
Dialister	0.0004	0.0001	0.0001	0.0000	0.0001	0.0000	0.0001	0.0000	0.0000	0.3711	0.3011	0.6462
Intestinimonas	0.0000	0.0003	0.0000	0.0000	0.0000	0.0000	0.0002	0.0006	0.0001	0.3780	0.4310	0.2005
Paraprevotella	0.0013	0.0009	0.0004	0.0005	0.0005	0.0008	0.0008	0.0005	0.0003	0.3902	0.3590	0.8541
Treponema	0.0009	0.0243	0.0009	0.0003	0.0013	0.0021	0.0078	0.0011	0.0009	0.3925	0.5416	0.4243
Eubacterium	0.0013	0.0019	0.0006	0.0004	0.0006	0.0014	0.0016	0.0016	0.0009	0.3962	0.8069	0.2077
Faecalibacterium	0.0002	0.0019	0.0002	0.0001	0.0001	0.0004	0.0004	0.0004	0.0002	0.4144	0.5215	0.3797
Chlamydia	0.0008	0.0005	0.0002	0.0002	0.0009	0.0018	0.0014	0.0001	0.0005	0.4366	0.6983	0.6713
Streptococcus	0.0003	0.0002	0.0002	0.0001	0.0002	0.0003	0.0003	0.0004	0.0002	0.4396	0.3636	0.2316
Dorea	0.0009	0.0001	0.0001	0.0001	0.0002	0.0002	0.0001	0.0004	0.0002	0.4533	0.6005	0.6150
Alloprevotella	0.0027	0.0004	0.0010	0.0002	0.0007	0.0013	0.0026	0.0017	0.0007	0.4576	0.7317	0.2012
Bifidobacterium	0.0000	0.0001	0.0000	0.0000	0.0001	0.0002	0.0001	0.0003	0.0001	0.4654	0.2677	0.6240
Burkholderia	0.0000	0.0001	0.0000	0.0000	0.0001	0.0001	0.0001	0.0000	0.0001	0.4821	0.1187	0.2943
Capnocytophaga	0.0003	0.0000	0.0000	0.0000	0.0002	0.0004	0.0002	0.0000	0.0001	0.4904	0.9240	0.4270
Subdoligranulum	0.0000	0.0004	0.0000	0.0000	0.0001	0.0000	0.0004	0.0001	0.0001	0.5005	0.7706	0.1879
Fusobacterium	0.0002	0.0001	0.0000	0.0000	0.0001	0.0001	0.0005	0.0006	0.0002	0.5276	0.0952	0.0651
Mycoplasma	0.0004	0.0001	0.0000	0.0001	0.0001	0.0001	0.0001	0.0001	0.0002	0.5368	0.6354	0.7791
Anaerovibrio	0.0006	0.0000	0.0001	0.0001	0.0005	0.0007	0.0003	0.0001	0.0001	0.5477	0.7613	0.3137
Succinivibrio	0.0032	0.0001	0.0001	0.0003	0.0087	0.0003	0.0009	0.0000	0.0001	0.5482	0.5161	0.3881
Bacteroides	0.0375	0.0307	0.0262	0.0170	0.0171	0.0430	0.0326	0.0214	0.0144	0.5693	0.2388	0.7886
Phascolarctobacterium	0.0022	0.0004	0.0005	0.0005	0.0011	0.0003	0.0022	0.0005	0.0005	0.5729	0.9589	0.5337
Spirochaeta	0.0003	0.0002	0.0000	0.0000	0.0000	0.0014	0.0001	0.0000	0.0001	0.5782	0.2765	0.4413
Succinatimonas	0.0065	0.0002	0.0002	0.0014	0.0014	0.0002	0.0017	0.0012	0.0002	0.5810	0.5815	0.9956
Sutterella	0.0001	0.0001	0.0000	0.0000	0.0002	0.0001	0.0001	0.0001	0.0001	0.5921	0.6987	0.7087
Barnesiella	0.0025	0.0003	0.0012	0.0010	0.0008	0.0050	0.0004	0.0004	0.0003	0.5932	0.2051	0.2477
Lachnoanaerobaculum	0.0000	0.0001	0.0001	0.0000	0.0001	0.0001	0.0000	0.0001	0.0000	0.6071	0.5583	0.3413
Dysgonomonas	0.0008	0.0003	0.0004	0.0001	0.0003	0.0007	0.0005	0.0003	0.0004	0.6120	0.7546	0.6930
Anaerophaga	0.0008	0.0001	0.0000	0.0000	0.0001	0.0004	0.0000	0.0002	0.0001	0.6275	0.5390	0.7939
Porphyromonas	0.0018	0.0006	0.0006	0.0004	0.0006	0.0013	0.0006	0.0006	0.0008	0.6284	0.4676	0.7815
Draconibacterium	0.0001	0.0000	0.0001	0.0000	0.0001	0.0003	0.0002	0.0007	0.0003	0.6530	0.0944	0.2087

Parabacteroides	0.0040	0.0013	0.0014	0.0009	0.0010	0.0032	0.0015	0.0012	0.0008	0.6779	0.3122	0.5222
Acetobacter	0.0000	0.0000	0.0000	0.0000	0.0000	0.0000	0.0001	0.0003	0.0001	0.6779	0.0247	0.0232
Desulfovibrio	0.0000	0.0002	0.0000	0.0000	0.0001	0.0000	0.0003	0.0001	0.0001	0.6869	0.5681	0.3040
Prevotella	0.1926	0.0960	0.2001	0.1930	0.1437	0.1962	0.0945	0.1005	0.0719	0.7145	0.0997	0.0097
Fibrobacter	0.0007	0.0112	0.0011	0.0050	0.0031	0.0007	0.0003	0.0000	0.0002	0.7186	0.2874	0.0876
Sunxiuqinia	0.0005	0.0001	0.0000	0.0000	0.0000	0.0004	0.0001	0.0000	0.0003	0.7589	0.7298	0.9794
Blautia	0.0001	0.0006	0.0002	0.0002	0.0002	0.0007	0.0004	0.0006	0.0002	0.7783	0.6711	0.9060
Marinifilum	0.0005	0.0001	0.0001	0.0000	0.0002	0.0007	0.0002	0.0005	0.0001	0.7888	0.9123	0.8420
Odoribacter	0.0021	0.0002	0.0003	0.0003	0.0006	0.0011	0.0008	0.0010	0.0004	0.7994	0.8847	0.7943
Enterococcus	0.0001	0.0004	0.0000	0.0000	0.0001	0.0005	0.0001	0.0000	0.0001	0.8120	0.3831	0.2953
Veillonella	0.0006	0.0001	0.0001	0.0003	0.0002	0.0000	0.0001	0.0000	0.0000	0.8122	0.3484	0.2041
Elusimicrobium	0.0000	0.0001	0.0000	0.0000	0.0001	0.0001	0.0002	0.0064	0.0001	0.8192	0.3503	0.3522
Acinetobacter	0.0000	0.0002	0.0000	0.0000	0.0001	0.0001	0.0009	0.0002	0.0008	0.8787	0.0785	0.0661
Oscillibacter	0.0005	0.0010	0.0004	0.0001	0.0003	0.0013	0.0026	0.0006	0.0004	0.9045	0.5018	0.4981
Bacillus	0.0005	0.0004	0.0001	0.0002	0.0003	0.0004	0.0003	0.0006	0.0003	0.9093	0.7410	0.6236
Tannerella	0.0018	0.0002	0.0005	0.0007	0.0006	0.0011	0.0004	0.0010	0.0003	0.9184	0.6372	0.4672
Prolixibacter	0.0000	0.0002	0.0001	0.0000	0.0002	0.0002	0.0002	0.0002	0.0002	0.9257	0.1916	0.1778
Akkermansia	0.0002	0.0004	0.0001	0.0000	0.0000	0.0006	0.0002	0.0003	0.0002	0.9407	0.8268	0.8545
Pseudomonas	0.0000	0.0003	0.0001	0.0001	0.0000	0.0003	0.0003	0.0002	0.0002	0.9591	0.3000	0.2871
Flavobacterium	0.0010	0.0003	0.0001	0.0001	0.0004	0.0009	0.0003	0.0003	0.0002	0.9683	0.4627	0.4908

Supplementary Table S4. Comparison of the change in rumen methanogens of three treatment groups.

Taxa	0 mg			52 mg			91 mg			T-test		
	0-1	0-2	0-3	52-1	52-2	52-3	91-1	91-2	91-3	0_52	0_91	52_91
Methanobrevibacter	0.0001	0.0004	0.0025	0.0002	0.0001	0.0004	0.0002	0.0024	0.0023	0.3753	0.5759	0.1234
methanogenic archaeon mixed culture ISO4-G1	0.0000	0.0000	0.0000	0.0000	0.0000	0.0007	0.0000	0.0000	0.0000	0.3838	0.8324	0.3904
Methanobacteria	0.0001	0.0004	0.0026	0.0002	0.0002	0.0004	0.0003	0.0027	0.0025	0.3966	0.5016	0.1152
Methanobacteriaceae	0.0001	0.0004	0.0026	0.0002	0.0002	0.0004	0.0003	0.0027	0.0025	0.3966	0.5016	0.1152
Methanobacteriales	0.0001	0.0004	0.0026	0.0002	0.0002	0.0004	0.0003	0.0027	0.0025	0.3966	0.5016	0.1152
Methanomassiliicoccaceae	0.0000	0.0001	0.0000	0.0000	0.0000	0.0013	0.0000	0.0000	0.0000	0.4003	0.3107	0.3642
Methanomassiliicoccales	0.0000	0.0001	0.0000	0.0000	0.0000	0.0013	0.0000	0.0000	0.0000	0.4003	0.3107	0.3642
Methanobrevibacter millerae	0.0000	0.0001	0.0017	0.0000	0.0001	0.0002	0.0001	0.0022	0.0016	0.4140	0.4463	0.1221
Methanomicrobiaceae	0.0011	0.0001	0.0000	0.0000	0.0000	0.0003	0.0000	0.0000	0.0000	0.4724	0.3442	0.4072
Methanobrevibacter sp. YE315	0.0000	0.0000	0.0004	0.0000	0.0000	0.0001	0.0000	0.0001	0.0001	0.5537	0.7546	0.4782
Methanomicrobiales	0.0014	0.0001	0.0000	0.0000	0.0000	0.0010	0.0000	0.0000	0.0000	0.7898	0.3484	0.3945
Methanomicrobia	0.0014	0.0001	0.0000	0.0000	0.0000	0.0014	0.0001	0.0004	0.0002	0.9443	0.5461	0.6236

Supplementary Table S5. Comparisons of the functions of the rumen bacterial microbiota in three treatment group.

taxa	0 mg			52 mg			91 mg			T-test		
	0-1	0-2	0-3	52-1	52-2	52-3	91-1	91-2	91-3	0_52	0_91	51_91
K1001100 Metabolism	2852	3246	2035	2314	3161	2735	2100	1918	1886	0.96	0.11	0.04
K2000011 Genetic Information Processing	1131	1146	830	1051	1414	1254	984	1020	950	0.24	0.65	0.08
Carbohydrate Metabolism	868	863	640	643	871	772	608	555	552	0.79	0.05	0.05
Amino Acid Metabolism	757	885	532	633	893	706	556	465	481	0.89	0.10	0.04
Nucleotide Metabolism	551	600	388	448	627	557	433	378	377	0.73	0.15	0.06
K2000016 Environmental Information Processing	500	549	387	475	700	592	437	496	477	0.24	0.87	0.15
Energy Metabolism	489	557	346	404	546	481	399	355	326	0.87	0.19	0.06
Replication and Repair	427	453	291	353	440	397	263	257	251	0.92	0.06	0.01
K2000013 Translation	409	453	265	367	489	434	386	375	315	0.46	0.79	0.16
K1000230 Purine metabolism	409	424	303	342	478	431	319	283	278	0.53	0.10	0.04
Metabolism of Cofactors and Vitamins	390	538	261	325	452	385	256	239	240	0.92	0.13	0.02
K1000240 Pyrimidine metabolism	375	416	252	294	400	366	282	246	250	0.93	0.16	0.05
Membrane Transport	322	371	211	252	340	263	181	186	179	0.78	0.07	0.02
K2000020 Cellular Processes	268	222	288	416	699	620	459	556	547	0.02	0.00	0.56
Glycan Biosynthesis and Metabolism	253	269	193	222	287	272	177	189	183	0.51	0.08	0.02
K1000970 Aminoacyl-tRNA biosynthesis	242	248	175	192	263	241	198	192	165	0.76	0.22	0.11
K1000500 Starch and sucrose metabolism	221	186	176	161	213	178	134	131	116	0.64	0.01	0.02
Folding, Sorting and Degradation	218	170	191	230	351	292	223	265	258	0.06	0.04	0.32
K1000250 Alanine, aspartate and glutamate metabolism	214	213	154	143	219	176	164	137	138	0.65	0.09	0.24
K1002010 ABC transporters	211	254	144	168	220	168	119	133	125	0.65	0.07	0.03
Lipid Metabolism	209	213	148	163	210	204	168	160	170	0.93	0.32	0.16
Metabolism of Other Amino Acids	193	229	152	159	218	183	113	117	114	0.88	0.03	0.01
K1003440 Homologous recombination	192	209	122	182	230	206	112	102	103	0.35	0.06	0.00
K1003430 Mismatch repair	187	187	134	146	188	173	99	107	94	0.99	0.02	0.01
Signal Transduction	180	181	177	225	361	326	256	309	298	0.04	0.00	0.73
K2000025 Organismal Systems	177	69	229	301	516	454	342	444	427	0.03	0.01	0.80
K1000520 Amino sugar and nucleotide sugar metabolism	170	206	115	126	172	164	161	148	135	0.76	0.60	0.73
K1003010 Ribosome	167	205	90	175	226	193	188	183	150	0.30	0.61	0.27
Cell Growth and Death	163	131	138	185	299	270	208	236	227	0.04	0.00	0.48
K1000330 Arginine and proline metabolism	152	158	95	136	192	163	108	103	101	0.33	0.20	0.02
K1003030 DNA replication	147	170	103	146	191	165	88	89	83	0.31	0.05	0.00
K1003420 Nucleotide excision repair	140	125	98	101	138	120	100	91	92	0.94	0.10	0.08
Biosynthesis of Other Secondary Metabolites	130	116	99	89	137	119	81	86	70	1.00	0.02	0.07
K2000034 Human Diseases	126	84	141	198	296	275	181	251	245	0.02	0.02	0.46

K1000910 Nitrogen metabolism	125	124	91	86	119	124	95	81	79	0.83	0.08	0.13
K1000052 Galactose metabolism	121	108	86	79	116	114	86	79	90	0.91	0.14	0.22
K1000190 Oxidative phosphorylation	117	138	85	109	150	132	116	106	95	0.43	0.67	0.14
Biosynthesis of Polyketides and Terpenoids	115	144	81	100	138	111	71	76	64	0.90	0.08	0.02
K1000010 Glycolysis / Gluconeogenesis	114	113	76	84	106	103	92	89	91	0.83	0.46	0.37
K1000270 Cysteine and methionine metabolism	113	150	82	97	150	104	90	72	69	0.94	0.14	0.09
K1000680 Methane metabolism	112	133	64	83	106	87	93	69	80	0.64	0.36	0.32
K1000550 Peptidoglycan biosynthesis	112	129	77	97	125	119	63	67	57	0.68	0.05	0.00
K1004112 Cell cycle - Caulobacter	111	127	67	80	117	100	71	56	57	0.90	0.10	0.03
Endocrine System	104	53	110	130	210	191	152	194	191	0.04	0.02	0.95
K1000290 Valine, leucine and isoleucine biosynthesis	104	106	80	99	137	97	77	72	79	0.41	0.07	0.06
K1000710 Carbon fixation in photosynthetic organisms	104	109	67	77	107	91	68	77	62	0.92	0.16	0.08
K1000620 Pyruvate metabolism	103	125	68	69	110	94	80	62	68	0.73	0.18	0.18
K1003018 RNA degradation	101	75	76	82	132	112	93	103	101	0.22	0.17	0.55
K1002020 Two-component system	101	159	57	67	108	98	90	73	76	0.67	0.43	0.45
K1003070 Bacterial secretion system	98	95	61	76	95	77	56	47	49	0.89	0.05	0.01
K1000400 Phenylalanine, tyrosine and tryptophan biosynthesis	97	119	58	83	111	79	61	41	52	0.99	0.10	0.03
K1003060 Protein export	95	94	65	73	98	80	46	53	52	0.94	0.03	0.01
K1000040 Pentose and glucuronate interconversions	93	90	57	61	80	78	51	41	46	0.62	0.05	0.02
K1000260 Glycine, serine and threonine metabolism	89	104	68	66	95	72	59	50	48	0.53	0.03	0.06
K1000051 Fructose and mannose metabolism	89	93	67	82	105	83	73	59	67	0.56	0.14	0.05
Xenobiotics Biodegradation and Metabolism	89	90	51	72	95	79	75	69	68	0.73	0.67	0.19
K1000020 Citrate cycle (TCA cycle)	86	87	66	62	91	80	57	44	48	0.86	0.02	0.04
Transport and Catabolism	81	34	120	179	290	258	177	227	235	0.02	0.01	0.48
K2000012 Transcription	79	70	85	102	138	132	112	125	127	0.02	0.00	0.84
K1000030 Pentose phosphate pathway	78	86	53	75	93	76	63	61	56	0.48	0.29	0.03
K1000670 One carbon pool by folate	74	95	49	67	90	67	52	39	37	0.90	0.10	0.02
K1000720 Carbon fixation pathways in prokaryotes	70	64	54	56	76	61	39	34	31	0.84	0.01	0.01
K1000770 Pantothenate and CoA biosynthesis	69	78	50	52	80	56	29	31	41	0.82	0.02	0.04
K1000300 Lysine biosynthesis	67	96	56	59	92	69	47	43	42	0.98	0.07	0.04
K1003410 Base excision repair	65	78	44	69	90	75	58	49	53	0.25	0.43	0.02
K1000460 Cyanoamino acid metabolism	64	55	57	39	54	47	30	32	27	0.08	0.00	0.02
K1003020 RNA polymerase	64	69	54	59	78	86	67	60	61	0.26	0.95	0.23
K1000600 Sphingolipid metabolism	63	44	44	47	64	67	41	41	46	0.37	0.31	0.06
K1000564 Glycerophospholipid metabolism	61	73	48	48	59	55	51	60	56	0.45	0.55	0.71
K1000511 Other glycan degradation	61	42	53	40	48	58	41	33	45	0.68	0.13	0.23
K1004910 Insulin signaling pathway	60	29	67	73	114	99	88	116	113	0.06	0.02	0.53

K1000900 Terpenoid backbone biosynthesis	59	78	43	45	67	51	35	37	33	0.66	0.07	0.04
K1000650 Butanoate metabolism	59	51	42	33	65	54	37	28	33	1.00	0.03	0.14
K1000860 Porphyrin and chlorophyll metabolism	55	89	40	56	60	65	54	40	33	0.95	0.29	0.05
Environmental Adaptation	54	29	65	117	172	158	96	137	126	0.01	0.01	0.23
K1000340 Histidine metabolism	54	75	31	42	65	47	30	27	32	0.90	0.14	0.04
K1000630 Glyoxylate and dicarboxylate metabolism	53	60	27	31	38	33	30	31	30	0.28	0.18	0.16
K1000521 Streptomycin biosynthesis	53	52	40	43	58	51	38	42	33	0.72	0.10	0.06
K1000760 Nicotinate and nicotinamide metabolism	53	71	42	41	62	59	31	42	34	0.91	0.10	0.07
K1000940 Phenylpropanoid biosynthesis	49	31	42	22	41	38	23	25	22	0.42	0.03	0.16
K1000983 Drug metabolism - other enzymes	49	43	24	41	50	37	40	40	33	0.66	0.91	0.33
Cell Communication	48	0	65	66	118	100	94	117	113	0.08	0.03	0.47
K03701 excinuclease ABC subunit A	48	41	32	26	33	35	37	27	30	0.17	0.18	1.00
K05349 beta-glucosidase [EC:3.2.1.21]	48	30	42	22	41	38	23	25	22	0.47	0.04	0.16
K1000640 Propanoate metabolism	46	57	26	44	60	43	39	30	26	0.60	0.31	0.06
K1000540 Lipopolysaccharide biosynthesis	46	60	34	50	62	43	35	42	31	0.62	0.26	0.07
Infectious Diseases	45	38	33	40	77	70	47	55	57	0.12	0.04	0.47
K1000450 Selenocompound metabolism	44	61	28	43	62	51	30	35	34	0.52	0.31	0.03
K01190 beta-galactosidase [EC:3.2.1.23]	40	25	30	32	39	41	22	22	27	0.34	0.16	0.01
K1004626 Plant-pathogen interaction	39	29	44	82	121	112	71	96	90	0.01	0.01	0.24
K01955 carbamoyl-phosphate synthase large subunit [EC:6.3.5.5]	38	26	23	25	32	21	30	18	20	0.62	0.34	0.53
Nervous System	37	0	54	61	103	89	87	112	108	0.06	0.02	0.28
K1000561 Glycerolipid metabolism	36	47	17	24	34	32	37	33	31	0.74	0.97	0.36
K1000730 Thiamine metabolism	35	55	19	20	29	31	22	17	21	0.43	0.20	0.15
K1000061 Fatty acid biosynthesis	35	53	20	33	46	40	30	18	23	0.74	0.29	0.04
Neurodegenerative Diseases	34	13	56	82	108	103	70	110	106	0.01	0.03	0.88
Immune System	34	12	52	52	90	82	81	106	104	0.06	0.01	0.19

Supplementary Table S6. EOC-associated KO pathways.

	0mg			52mg				
taxa	0-1	0-2	0-3	52-1	52-2	52-3	T-test	FDR
K2000020 Cellular Processes	268	222	288	416	699	620	0.021092957	0.034719627
K2000025 Organismal Systems	177	69	229	301	516	454	0.028776583	0.03847304
K2000034 Human Diseases	126	84	141	198	296	275	0.015363552	0.030523093
K2000012 Transcription	79	70	85	102	138	132	0.018354004	0.032350931
K1004626 Plant-pathogen interaction	39	29	44	82	121	112	0.00578517	0.030523093
K2000035 Cancers	31	12	45	59	84	78	0.021936618	0.035502684
K1005200 Pathways in cancer	29	12	40	44	68	61	0.047163492	0.048606172
K1004914 Progesterone-mediated oocyte maturation	29	12	31	44	77	69	0.027685954	0.037628424
K1004070 Phosphatidylinositol signaling system	28	18	39	42	58	54	0.041061191	0.045500239
K1004144 Endocytosis	28	0	55	97	163	136	0.013771702	0.030523093
K1004114 Oocyte meiosis	27	0	39	63	95	85	0.01672169	0.030698028
K00688 starch phosphorylase [EC:2.4.1.1]	26	22	19	11	11	9	0.004920932	0.030523093
K1004142 Lysosome	25	11	32	40	61	67	0.031337788	0.040838257
K01006 pyruvate, orthophosphate dikinase [EC:2.7.9.1]	23	24	17	12	12	17	0.049354377	0.049354377
K1005214 Glioma	19	0	26	35	41	38	0.044558795	0.048076595
K1004530 Tight junction	19	0	27	34	48	42	0.044257673	0.04796206
K1004340 Hedgehog signaling pathway	18	1	27	43	70	63	0.017564621	0.0317713
K1004150 mTOR signaling pathway	17	0	28	32	56	48	0.048070274	0.048979839
K13412 calcium-dependent protein kinase [EC:2.7.11.1]	17	0	27	48	84	72	0.015579542	0.030523093
K01740 O-acetylhomoserine (thiol)-lyase [EC:2.5.1.49]	16	14	16	7	12	2	0.048183419	0.048979839
K1004722 Neurotrophin signaling pathway	16	0	23	32	36	35	0.036704115	0.044699071
K1004113 Meiosis - yeast	16	0	23	31	60	51	0.03494535	0.044020152
K1004270 Vascular smooth muscle contraction	16	0	21	31	47	41	0.025480198	0.035951532
K1004720 Long-term potentiation	16	0	22	36	50	44	0.016486323	0.03058205
K1005010 Alzheimer's disease	15	8	16	25	27	27	0.006879081	0.030523093
K1004111 Cell cycle - yeast	14	2	21	33	57	53	0.01889863	0.032520746
K1004120 Ubiquitin mediated proteolysis	13	0	32	42	67	47	0.037067686	0.044796295
K1003040 Spliceosome	13	1	26	33	50	39	0.03562386	0.044037535
K1004010 MAPK signaling pathway	13	0	18	24	38	32	0.035430854	0.044020152
K1004916 Melanogenesis	12	0	16	26	38	33	0.017910703	0.031927775
K1004912 GnRH signaling pathway	12	0	18	31	40	37	0.011738602	0.030523093
K03702 excinuclease ABC subunit B	11	13	9	8	6	6	0.031375734	0.040838257
K01785 aldose 1-epimerase [EC:5.1.3.3]	11	11	11	9	5	7	0.025721421	0.035951532
K00820 glucosamine--fructose-6-phosphate aminotransferase (isomerizing) [EC:2.6.1.16]	11	15	10	6	7	5	0.021311641	0.034719627
K1004110 Cell cycle	11	0	19	44	85	77	0.012835042	0.030523093
K1005016 Huntington's disease	11	4	26	42	61	57	0.010311896	0.030523093
K1004612 Antigen processing and presentation	11	12	12	22	29	29	0.003125589	0.030523093

K2000032 Development	10	0	13	16	28	27	0.04365947	0.04752314
K1004711 Circadian rhythm - fly	10	0	15	22	32	32	0.021233063	0.034719627
K1000510 N-Glycan biosynthesis	10	14	14	19	24	25	0.011912539	0.030523093
K1004115 p53 signaling pathway	9	0	15	18	43	40	0.046400593	0.048606172
K1005340 Primary immunodeficiency	9	8	5	11	16	12	0.043439495	0.047493848
K01847 methylmalonyl-CoA mutase [EC:5.4.99.2]	9	8	3	12	13	12	0.039737619	0.04486632
K1004320 Dorso-ventral axis formation	9	0	10	14	21	21	0.035284851	0.044020152
K1004962 Vasopressin-regulated water reabsorption	9	0	23	34	65	47	0.027497111	0.037579385
K1000908 Zeatin biosynthesis	9	12	8	14	17	14	0.026997302	0.037258448
K00791 tRNA dimethylallyltransferase [EC:2.5.1.75]	9	12	8	14	17	14	0.026997302	0.037258448
K03601 exodeoxyribonuclease VII large subunit [EC:3.1.11.6]	9	10	7	4	6	5	0.025386448	0.035951532
K02218 casein kinase 1 [EC:2.7.11.1]	9	0	12	21	27	28	0.012174924	0.030523093
K01206 alpha-L-fucosidase [EC:3.2.1.51]	9	9	10	2	3	6	0.010469669	0.030523093
K01776 glutamate racemase [EC:5.1.1.3]	9	8	9	7	7	7	0.007490434	0.030523093
K03572 DNA mismatch repair protein MutL	8	10	5	3	4	2	0.040545533	0.04523961
K01687 dihydroxy-acid dehydratase [EC:4.2.1.9]	8	6	8	1	5	1	0.028460203	0.038257978
K03110 fused signal recognition particle receptor	8	9	6	3	5	2	0.025481481	0.035951532
K1004020 Calcium signaling pathway	8	0	14	22	28	25	0.016044187	0.030523093
K1004740 Olfactory transduction	8	0	14	26	44	38	0.012645619	0.030523093
K1005219 Bladder cancer	7	0	7	10	13	12	0.048502075	0.049010782
K01613 phosphatidylserine decarboxylase [EC:4.1.1.65]	7	10	8	5	4	2	0.020092107	0.033853824
K01126 glycerophosphoryl diester phosphodiesterase [EC:3.1.4.46]	6	5	3	2	2	2	0.039020527	0.04486632
K1003050 Proteasome	6	1	11	21	35	38	0.013313327	0.030523093
K04041 fructose-1,6-bisphosphatase III [EC:3.1.3.11]	5	3	7	10	12	8	0.037590112	0.04486632
K1005110 Vibrio cholerae infection	5	0	6	13	35	32	0.032153283	0.04163004
K01939 adenylosuccinate synthase [EC:6.3.4.4]	5	4	4	2	3	3	0.024110111	0.035951532
K07891 Ras-related protein Rab-22	5	0	9	15	26	20	0.018904336	0.032520746
K00928 aspartate kinase [EC:2.7.2.4]	5	5	6	4	4	4	0.01613009	0.030523093
K01443 N-acetylglucosamine-6-phosphate deacetylase [EC:3.5.1.25]	5	5	3	1	2	1	0.015799848	0.030523093
K01686 mannonate dehydratase [EC:4.2.1.8]	5	4	5	7	9	9	0.007933092	0.030523093
K02112 F-type H+-transporting ATPase subunit beta [EC:3.6.3.14]	5	2	3	10	9	9	0.003125589	0.030523093
K06269 serine/threonine-protein phosphatase PP1 catalytic subunit [EC:3.1.3.16]	4	0	7	8	17	14	0.048811551	0.049010782
K00625 phosphate acetyltransferase [EC:2.3.1.8]	4	6	3	2	2	1	0.047420656	0.048606172
K02945 small subunit ribosomal protein S1	4	6	2	7	10	8	0.040642089	0.04523961
K00029 malate dehydrogenase (oxaloacetate-decarboxylating)(NADP+) [EC:1.1.1.40]	4	4	1	0	0	0	0.039941968	0.04486632
K02950 small subunit ribosomal protein S12	4	2	3	5	7	5	0.039020527	0.04486632
K07897 Ras-related protein Rab-7A	4	0	5	7	10	10	0.030319669	0.040100207
K01770 2-C-methyl-D-erythritol 2,4-cyclodiphosphate synthase [EC:4.6.1.12]	4	8	5	1	2	1	0.025481481	0.035951532
K01881 prolyl-tRNA synthetase [EC:6.1.1.15]	4	4	3	5	5	6	0.024110111	0.035951532
K04568 lysyl-tRNA synthetase, class II [EC:6.1.1.6]	4	5	3	1	2	0	0.021311641	0.034719627

K01424 L-asparaginase [EC:3.5.1.1]	4	6	5	3	2	2	0.01613009	0.030523093
K01711 GDPmannose 4,6-dehydratase [EC:4.2.1.47]	4	3	2	6	8	8	0.007966202	0.030523093
K02232 adenosylcobyric acid synthase [EC:6.3.5.10]	4	5	4	2	1	2	0.004812678	0.030523093
K11839 ubiquitin carboxyl-terminal hydrolase 8 [EC:3.4.19.12]	4	0	5	16	25	23	0.0042248	0.030523093
K00793 riboflavin synthase [EC:2.5.1.9]	4	4	4	7	6	6	0.00219213	0.030523093
K02495 oxygen-independent coproporphyrinogen III oxidase [EC:1.3.99.22]	3	6	2	7	8	7	0.042394467	0.046766991
K00651 homoserine O-succinyltransferase [EC:2.3.1.46]	3	2	5	6	10	8	0.032532525	0.04190053
K03786 3-dehydroquinate dehydratase II [EC:4.2.1.10]	3	2	1	4	5	4	0.024896163	0.035951532
K01619 deoxyribose-phosphate aldolase [EC:4.1.2.4]	3	4	3	2	2	1	0.024110111	0.035951532
K07904 Ras-related protein Rab-11A	3	0	13	19	29	25	0.017730122	0.031836569
K1004140 Regulation of autophagy	3	0	12	20	37	33	0.016316452	0.03058205
K05868 cyclin B	3	0	3	10	22	19	0.016010966	0.030523093
K1004130 SNARE interactions in vesicular transport	3	0	7	12	19	15	0.013863227	0.030523093
K1004710 Circadian rhythm	3	0	4	9	16	12	0.0132356	0.030523093
K10408 dynein heavy chain, axonemal	3	0	11	19	22	25	0.009520176	0.030523093
K12619 5'-3' exoribonuclease 2 [EC:3.1.13.-]	3	0	4	9	10	10	0.004181072	0.030523093
K11253 histone H3	2	0	3	4	11	9	0.048742093	0.049010782
K02227 adenosylcobinamide-phosphate synthase [EC:6.3.1.10]	2	2	1	1	0	0	0.047420656	0.048606172
K10117 raffinose/stachyose/melibiose transport system substrate-binding protein	2	2	1	0	1	0	0.047420656	0.048606172
K00074 3-hydroxybutyryl-CoA dehydrogenase [EC:1.1.1.157]	2	1	2	0	1	0	0.047420656	0.048606172
K07466 replication factor A1	2	0	2	3	4	5	0.039020527	0.04486632
K03120 transcription initiation factor TFIID TATA-box-binding protein	2	0	4	7	6	5	0.036277825	0.044399726
K03259 translation initiation factor 4E	2	0	3	5	13	11	0.035371373	0.044020152
K02156 aubergine	2	0	3	5	11	11	0.028437314	0.038257978
K1003022 Basal transcription factors	2	0	5	10	10	7	0.019441768	0.032983965
K1005322 Systemic lupus erythematosus	2	0	4	7	13	10	0.018411099	0.032350931
K08957 casein kinase 1, alpha [EC:2.7.11.1]	2	0	3	7	15	13	0.017457615	0.0317713
K01575 acetolactate decarboxylase [EC:4.1.1.5]	2	1	1	0	0	0	0.01613009	0.030523093
K03230 type III secretion protein V	2	1	1	0	0	0	0.01613009	0.030523093
K02063 thiamine transport system permease protein	2	1	1	0	0	0	0.01613009	0.030523093
K07151 dolichyl-diphosphooligosaccharide--protein glycosyltransferase [EC:2.4.99.18]	2	2	2	0	1	1	0.01613009	0.030523093
K1005410 Hypertrophic cardiomyopathy (HCM)	2	0	6	10	12	10	0.0132356	0.030523093
K11927 ATP-dependent RNA helicase RhlE [EC:3.6.4.13]	2	2	1	0	0	0	0.007490434	0.030523093
K01629 rhamnulose-1-phosphate aldolase [EC:4.1.2.19]	2	2	1	0	0	0	0.007490434	0.030523093
K06631 polo-like kinase 1 [EC:2.7.11.21]	2	0	3	7	9	8	0.003862848	0.030523093
K00147 glutamate-5-semialdehyde dehydrogenase [EC:1.2.1.41]	2	4	2	7	8	7	0.00331976	0.030523093
K00657 diamine N-acetyltransferase [EC:2.3.1.57]	2	1	1	5	6	6	0.000777907	0.030523093
K13043 N-succinyl-L-ornithine transcarbamylase [EC:2.1.3.11]	1	1	3	5	3	5	0.047420656	0.048606172
K06674 structural maintenance of chromosome 2	1	1	2	3	3	2	0.047420656	0.048606172
K07198 5'-AMP-activated protein kinase, catalytic alpha subunit [EC:2.7.11.11]	1	0	5	6	7	8	0.037590112	0.04486632

K12581 CCR4-NOT transcription complex subunit 7/8	1	0	4	6	16	12	0.037148147	0.044796295
K07359 calcium/calmodulin-dependent protein kinase kinase [EC:2.7.11.17]	1	0	2	3	7	5	0.036277825	0.044399726
K12492 ADP-ribosylation factor GTPase-activating protein 1	1	0	2	6	4	3	0.034109423	0.043476259
K03006 DNA-directed RNA polymerase II subunit RPB1 [EC:2.7.7.6]	1	0	2	3	6	4	0.034109423	0.043476259
K1005130 Pathogenic Escherichia coli infection	1	0	2	3	7	6	0.031375734	0.040838257
K1005217 Basal cell carcinoma	1	0	6	8	11	9	0.027110822	0.037258448
K04802 proliferating cell nuclear antigen	1	0	0	2	5	5	0.025386448	0.035951532
K02206 cyclin-dependent kinase 2 [EC:2.7.11.22]	1	0	2	3	6	5	0.025386448	0.035951532
K01373 cathepsin F [EC:3.4.22.41]	1	0	2	3	4	5	0.021311641	0.034719627
K07889 Ras-related protein Rab-5C	1	0	2	7	14	8	0.018562565	0.032385752
K03347 cullin 1	1	0	1	2	2	2	0.01613009	0.030523093
K13829 shikimate kinase / 3-dehydroquinate synthase [EC:2.7.1.71 4.2.3.4]	1	1	1	2	2	3	0.01613009	0.030523093
K00068 sorbitol-6-phosphate 2-dehydrogenase [EC:1.1.1.140]	1	2	1	0	0	0	0.01613009	0.030523093
K08515 vesicle-associated membrane protein 7	1	0	1	3	6	5	0.0132356	0.030523093
K12194 charged multivesicular body protein 4	1	0	1	2	3	3	0.0132356	0.030523093
K01195 beta-glucuronidase [EC:3.2.1.31]	1	2	2	3	4	4	0.0132356	0.030523093
K1000944 Flavone and flavonol biosynthesis	1	2	2	3	4	4	0.0132356	0.030523093
K01369 legumain [EC:3.4.22.34]	1	0	1	2	3	3	0.0132356	0.030523093
K00053 ketol-acid reductoisomerase [EC:1.1.1.86]	1	1	0	7	8	4	0.010469669	0.030523093
K02109 F-type H+-transporting ATPase subunit b	1	3	1	5	6	5	0.007933092	0.030523093
K06630 14-3-3 protein epsilon	1	0	2	4	4	5	0.007490434	0.030523093
K03283 heat shock 70kDa protein 1/8	1	0	3	6	9	8	0.007090812	0.030523093
K06689 ubiquitin-conjugating enzyme E2 D/E [EC:6.3.2.19]	1	0	7	14	20	17	0.006793715	0.030523093
K07258 D-alanyl-D-alanine carboxypeptidase (penicillin-binding protein 5/6) [EC:3.4.16.4]	1	1	1	3	5	4	0.006533376	0.030523093
K07827 GTPase KRas	1	0	2	4	5	5	0.005328128	0.030523093
K02183 calmodulin	1	0	3	15	12	14	0.000586891	0.030523093
K06109 Ras-related protein Rab-13	1	0	1	10	9	11	0.000151011	0.02485092
K02389 flagellar basal-body rod modification protein FlgD	0	1	0	1	2	2	0.047420656	0.048606172
K10756 replication factor C subunit 3/5	0	0	1	1	2	2	0.047420656	0.048606172
K02727 20S proteasome subunit alpha 7 [EC:3.4.25.1]	0	0	1	1	2	2	0.047420656	0.048606172
K10839 UV excision repair protein RAD23	0	0	1	1	2	2	0.047420656	0.048606172
K01920 glutathione synthase [EC:6.3.2.3]	0	0	0	1	4	4	0.039941968	0.04486632
K12196 vacuolar protein-sorting-associated protein 4	0	0	0	2	5	2	0.039941968	0.04486632
K08959 casein kinase 1, delta [EC:2.7.11.1]	0	0	0	1	4	4	0.039941968	0.04486632
K02901 large subunit ribosomal protein L27e	0	0	0	1	4	4	0.039941968	0.04486632
K02434 aspartyl-tRNA(Asn)/glutamyl-tRNA(Gln) amidotransferase subunit B [EC:6.3.5.6 6.3.5.7]	0	0	0	1	4	3	0.039020527	0.04486632
K12575 kinetochore protein SLK19	0	0	0	1	3	4	0.039020527	0.04486632
K08341 GABA(A) receptor-associated protein	0	0	1	2	4	2	0.035168453	0.044020152
K01878 glycyl-tRNA synthetase alpha chain [EC:6.1.1.14]	0	0	0	2	3	1	0.025721421	0.035951532
K01634 sphinganine-1-phosphate aldolase [EC:4.1.2.27]	0	0	0	1	2	3	0.025721421	0.035951532

K03013 DNA-directed RNA polymerases I, II, and III subunit RPABC1	0	0	0	1	3	2	0.025721421	0.035951532
K03063 26S proteasome regulatory subunit T3	0	0	0	1	3	2	0.025721421	0.035951532
K02542 DNA replication licensing factor MCM6 [EC:3.6.4.12]	0	0	0	1	2	3	0.025721421	0.035951532
K12670 oligosaccharyltransferase complex subunit beta	0	0	0	2	1	3	0.025721421	0.035951532
K07888 Ras-related protein Rab-5B	0	0	0	1	2	3	0.025721421	0.035951532
K1004622 RIG-I-like receptor signaling pathway	0	0	1	3	7	4	0.025481481	0.035951532
K12879 THO complex subunit 2	0	0	0	1	3	3	0.024896163	0.035951532
K10643 CCR4-NOT transcription complex subunit 4 [EC:6.3.2.19]	0	0	0	2	6	6	0.024896163	0.035951532
K02728 20S proteasome subunit alpha 3 [EC:3.4.25.1]	0	0	0	1	3	3	0.024896163	0.035951532
K02885 large subunit ribosomal protein L19e	0	0	0	1	3	3	0.024896163	0.035951532
K01962 acetyl-CoA carboxylase carboxyl transferase subunit alpha [EC:6.4.1.2]	0	0	0	1	3	3	0.024896163	0.035951532
K08991 crossover junction endonuclease MUS81 [EC:3.1.22.-]	0	0	2	3	5	4	0.019441768	0.032983965
K03260 translation initiation factor 4G	0	1	0	2	4	3	0.01613009	0.030523093
K04710 ceramide synthetase [EC:2.3.1.24]	0	0	0	1	1	2	0.01613009	0.030523093
K03064 26S proteasome regulatory subunit T4	0	0	0	1	2	1	0.01613009	0.030523093
K08734 DNA mismatch repair protein MLH1	0	0	0	1	1	2	0.01613009	0.030523093
K02936 large subunit ribosomal protein L7Ae	0	0	0	1	1	2	0.01613009	0.030523093
K12666 oligosaccharyltransferase complex subunit alpha (ribophorin I)	0	0	0	2	5	5	0.01613009	0.030523093
K03037 26S proteasome regulatory subunit N7	0	0	0	1	2	1	0.01613009	0.030523093
K12857 Prp8 binding protein	0	0	0	2	1	1	0.01613009	0.030523093
K02145 V-type H+-transporting ATPase subunit A [EC:3.6.3.14]	0	0	0	1	1	2	0.01613009	0.030523093
K08506 syntaxin of plants SYP7	0	0	0	1	1	2	0.01613009	0.030523093
K12251 N-carbamoylputrescine amidase [EC:3.5.1.53]	0	1	0	4	3	2	0.01613009	0.030523093
K12195 charged multivesicular body protein 6	0	0	0	1	2	1	0.01613009	0.030523093
K02900 large subunit ribosomal protein L27Ae	0	0	0	1	1	2	0.01613009	0.030523093
K02947 small subunit ribosomal protein S10e	0	0	0	1	1	2	0.01613009	0.030523093
K05755 actin related protein 2/3 complex, subunit 4	0	0	0	1	1	2	0.01613009	0.030523093
K05692 actin beta/gamma 1	0	0	0	1	2	1	0.01613009	0.030523093
K1005412 Arrhythmogenic right ventricular cardiomyopathy (ARVC)	0	0	0	1	2	1	0.01613009	0.030523093
K02932 large subunit ribosomal protein L5e	0	0	0	1	1	2	0.01613009	0.030523093
K08333 phosphoinositide-3-kinase, regulatory subunit 4 [EC:2.7.11.1]	0	0	0	1	2	1	0.01613009	0.030523093
K12275 translocation protein SEC62	0	0	0	1	1	2	0.01613009	0.030523093
K12837 splicing factor U2AF 65 kDa subunit	0	0	0	1	1	2	0.01613009	0.030523093
K02940 large subunit ribosomal protein L9e	0	0	0	1	1	2	0.01613009	0.030523093
K01108 phosphatidylinositol-3-phosphatase [EC:3.1.3.64]	0	0	0	1	2	1	0.01613009	0.030523093
K01078 acid phosphatase [EC:3.1.3.2]	0	0	0	2	5	4	0.014172595	0.030523093
K10901 bloom syndrome protein [EC:3.6.4.12]	0	0	1	6	3	4	0.0132356	0.030523093
K06228 fused [EC:2.7.11.1]	0	0	3	7	6	5	0.012348477	0.030523093
K03094 S-phase kinase-associated protein 1	0	0	1	3	5	3	0.011056493	0.030523093
K01363 cathepsin B [EC:3.4.22.1]	0	0	1	3	6	5	0.01005971	0.030523093

K00888 phosphatidylinositol 4-kinase [EC:2.7.1.67]	0	0	0	3	6	4	0.007966202	0.030523093
K08486 syntaxin 1B/2/3	0	0	1	2	3	3	0.007762603	0.030523093
K1004614 Renin-angiotensin system	0	1	0	3	2	3	0.007762603	0.030523093
K00921 1-phosphatidylinositol-3-phosphate 5-kinase [EC:2.7.1.150]	0	0	1	2	3	3	0.007762603	0.030523093
K12830 splicing factor 3B subunit 3	0	0	0	1	2	2	0.007490434	0.030523093
K02958 small subunit ribosomal protein S15e	0	0	0	1	2	2	0.007490434	0.030523093
K09540 translocation protein SEC63	0	0	1	2	2	2	0.007490434	0.030523093
K02729 20S proteasome subunit alpha 5 [EC:3.4.25.1]	0	0	0	1	2	2	0.007490434	0.030523093
K00694 cellulose synthase (UDP-forming) [EC:2.4.1.12]	0	0	0	1	2	2	0.007490434	0.030523093
K08281 nicotinamidase/pyrazinamidase [EC:3.5.1.19 3.5.1.-]	0	0	0	1	2	2	0.007490434	0.030523093
K03124 transcription initiation factor TFIIB	0	0	0	1	2	2	0.007490434	0.030523093
K07203 FKBP12-rapamycin complex-associated protein	0	0	0	4	4	2	0.007490434	0.030523093
K10949 ER lumen protein retaining receptor	0	0	0	2	1	2	0.007490434	0.030523093
K12184 ESCRT-I complex subunit VPS28	0	0	0	1	2	2	0.007490434	0.030523093
K12614 ATP-dependent RNA helicase DDX6/DHH1 [EC:3.6.4.13]	0	0	0	1	2	2	0.007490434	0.030523093
K02898 large subunit ribosomal protein L26e	0	0	0	1	2	2	0.007490434	0.030523093
K11481 aurora kinase A [EC:2.7.11.1]	0	0	0	1	2	2	0.007490434	0.030523093
K01839 phosphopentomutase [EC:5.4.2.7]	0	0	0	1	2	2	0.007490434	0.030523093
K06210 nicotinamide mononucleotide adenylyltransferase [EC:2.7.7.1 2.7.7.18]	0	0	0	2	1	2	0.007490434	0.030523093
K02955 small subunit ribosomal protein S14e	0	0	0	1	2	2	0.007490434	0.030523093
K04532 amyloid beta precursor protein binding protein 1	0	0	0	1	2	2	0.007490434	0.030523093
K03036 26S proteasome regulatory subunit N6	0	0	0	1	2	2	0.007490434	0.030523093
K10573 ubiquitin-conjugating enzyme E2 A [EC:6.3.2.19]	0	0	0	2	2	1	0.007490434	0.030523093
K11423 histone-lysine N-methyltransferase SETD2 [EC:2.1.1.43]	0	0	0	2	1	2	0.007490434	0.030523093
K10546 putative multiple sugar transport system substrate-binding protein	0	0	0	1	2	2	0.007490434	0.030523093
K01254 leukotriene-A4 hydrolase [EC:3.3.2.6]	0	0	0	1	2	2	0.007490434	0.030523093
K05759 profilin	0	0	0	1	2	2	0.007490434	0.030523093
K01120 3',5'-cyclic-nucleotide phosphodiesterase [EC:3.1.4.17]	0	0	0	2	4	3	0.006533376	0.030523093
K06675 structural maintenance of chromosome 4	0	0	0	2	3	4	0.006533376	0.030523093
K1005416 Viral myocarditis	0	1	1	6	7	4	0.006073612	0.030523093
K08269 serine/threonine-protein kinase ULK/ATG1 [EC:2.7.11.1]	0	0	4	10	14	12	0.003772017	0.030523093
K02155 V-type H+-transporting ATPase 16kDa proteolipid subunit	0	0	0	3	4	5	0.002278426	0.030523093
K10742 DNA replication ATP-dependent helicase Dna2 [EC:3.6.4.12]	0	0	0	2	2	3	0.00219213	0.030523093
K03407 two-component system, chemotaxis family, sensor kinase CheA [EC:2.7.13.3]	0	0	0	2	3	2	0.00219213	0.030523093
K13289 cathepsin A (carboxypeptidase C) [EC:3.4.16.5]	0	0	0	3	2	2	0.00219213	0.030523093
K10798 poly [ADP-ribose] polymerase [EC:2.4.2.30]	0	0	0	3	2	2	0.00219213	0.030523093
K04460 serine/threonine-protein phosphatase 5 [EC:3.1.3.16]	0	0	0	2	3	3	0.001323897	0.030523093
K12603 CCR4-NOT transcription complex subunit 6 [EC:3.1.13.4]	0	0	0	4	4	5	0.00020204	0.02485092
